# (*E*)-2-[(2,4-Dichloro­phen­yl)imino­meth­yl]benzene-1,4-diol monohydrate

**DOI:** 10.1107/S1600536809045103

**Published:** 2009-11-07

**Authors:** Zarife Sibel Şahin, Sūmeyye Gūmūş, Mustafa Macit, Şamil Işık

**Affiliations:** a Department of Physics, Faculty of Arts and Sciences, Ondokuz Mayıs University, Kurupelit, TR-55139 Samsun, Turkey; b Department of Chemistry, Faculty of Arts and Sciences, Ondokuz Mayıs University, TR-55139 Samsun, Turkey

## Abstract

The title compound, C_13_H_9_Cl_2_NO_2_·H_2_O, represents a Schiff base which adopts the phenol–imine tautomeric form in the solid state. The mol­ecule is approximately planar (r.m.s. deviation 0.0818 Å), and the dihedral angle between the two aromatic rings is 7.46 (12)°. An O—H⋯N inter­action generates an *S*(6) ring. In the crystal, mol­ecules are linked by inter­molecular O—H⋯O hydrogen bonds involving the solvent water mol­ecule, forming chains.

## Related literature

For the biological properties of Schiff bases see: Lozier *et al.* (1975 ▶), Dao *et al.* (2000 ▶). For the coordination chemistry of Schiff bases see: Kargar *et al.* (2009 ▶); Yeap *et al.* (2009 ▶). For a discussion of Schiff bases tautomerism, see: Şahin *et al.* (2005 ▶); Hadjoudis *et al.* (1987 ▶). For a related structure, see: Zhang (2009 ▶).
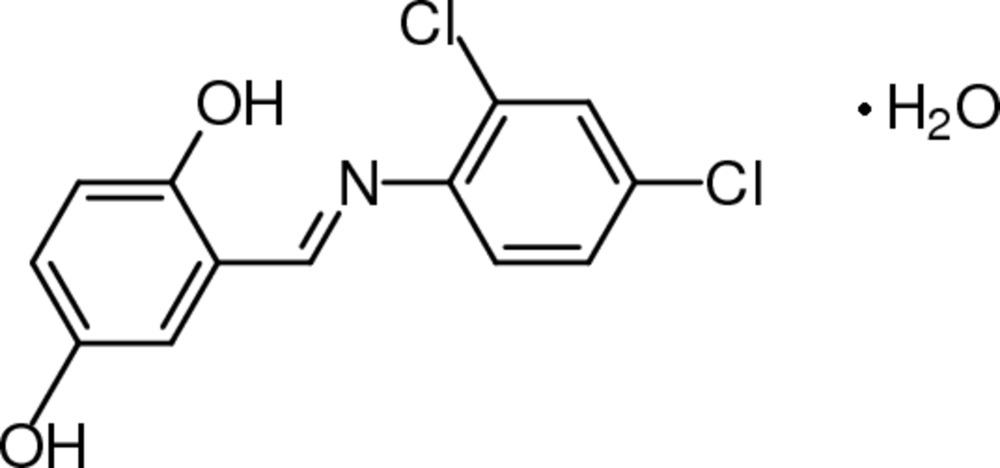


## Experimental

### 

#### Crystal data

C_13_H_9_Cl_2_NO_2_·H_2_O
*M* *_r_* = 300.13
Monoclinic, 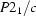

*a* = 4.6899 (2) Å
*b* = 17.4289 (6) Å
*c* = 16.1645 (7) Å
β = 95.923 (3)°
*V* = 1314.23 (9) Å^3^
*Z* = 4
Mo *K*α radiation
μ = 0.50 mm^−1^
*T* = 296 K
0.90 × 0.56 × 0.25 mm

#### Data collection

Stoe IPDS II diffractometer
Absorption correction: integration (*X-RED32*; Stoe & Cie, 2002 ▶) *T*_min_ = 0.801, *T*_max_ = 0.959
11982 measured reflections
2585 independent reflections
1879 reflections with *I* > 2σ(*I*)
*R*_int_ = 0.050

#### Refinement

*R*[*F* ^2^ > 2σ(*F* ^2^)] = 0.037
*wR*(*F* ^2^) = 0.098
*S* = 0.97
2585 reflections
188 parameters
H atoms treated by a mixture of independent and constrained refinement
Δρ_max_ = 0.14 e Å^−3^
Δρ_min_ = −0.29 e Å^−3^

### 

Data collection: *X-AREA* (Stoe & Cie, 2002 ▶); cell refinement: *X-AREA*; data reduction: *X-RED32*; program(s) used to solve structure: *SHELXS97* (Sheldrick, 2008 ▶); program(s) used to refine structure: *SHELXL97* (Sheldrick, 2008 ▶); molecular graphics: *ORTEP-3 for Windows* (Farrugia, 1997 ▶); software used to prepare material for publication: *WinGX* (Farrugia, 1999 ▶).

## Supplementary Material

Crystal structure: contains datablocks I, global. DOI: 10.1107/S1600536809045103/bh2254sup1.cif

Structure factors: contains datablocks I. DOI: 10.1107/S1600536809045103/bh2254Isup2.hkl

Additional supplementary materials:  crystallographic information; 3D view; checkCIF report

## Figures and Tables

**Table 1:** Hydrogen-bond geometry (Å, °)

*D*—H⋯*A*	*D*—H	H⋯*A*	*D*⋯*A*	*D*—H⋯*A*
O3—H1*O*⋯O2^i^	0.85 (4)	1.94 (4)	2.774 (3)	170 (3)
O2—H2⋯N1	0.87 (4)	1.77 (3)	2.569 (2)	152 (3)
O3—H2*O*⋯O1^ii^	0.82 (4)	2.49 (5)	3.184 (3)	143 (4)
O1—H1⋯O3	0.87 (4)	1.79 (4)	2.659 (3)	171 (3)

Symmetry codes: (i) 
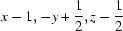
; (ii) 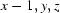.

**Fig. 1.**
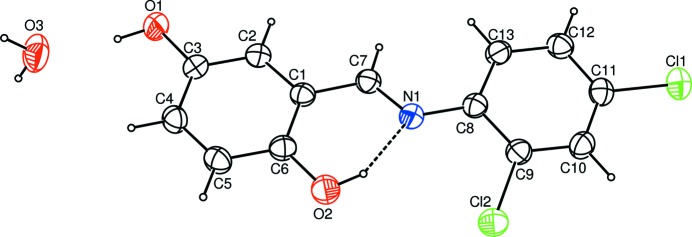
The molecular structure of the title compound, showing the atom-numbering scheme. Displacement ellipsoids are drawn at the 30% probability level.

**Fig. 2.**
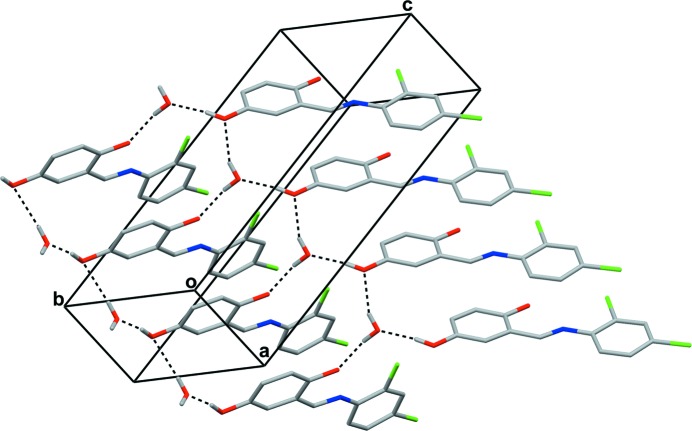
Part of the crystal structure, showing the formation R66(22) rings. Hydrogen bonds are indicated by dashed lines. H atoms not involved in these interactions have been omitted for clarity.

## supplementary crystallographic information

### Comment

Schiff bases often exhibit various biological activities and in many cases were shown to have antibacterial, anticancer, anti-inflammatory and antitoxic properties (Lozier *et al.*, 1975; Dao *et al.*, 2000). Schiff bases have also been used as versatile ligands in coordination chemistry (Kargar *et al.*, 2009; Yeap *et al.*, 2009). There are two types of intramolecular hydrogen bonds in Schiff bases, which may be stabilized either in keto-amine (N—H···O hydrogen bond) (Şahin *et al.*, 2005) or phenol-imine (N···H—O hydrogen bond) tautomeric forms (Hadjoudis *et al.*, 1987). The present X-ray investigation shows that the title compound is a Schiff base and exists in the phenol-imine form in the solid-state.

An *ORTEP-3* (Farrugia, 1997) plot and crystal packing of the molecule of the title compound are shown in Figs. 1 and 2, respectively. The molecule is approximately planar. The dihedral angle between the two aromatic rings is 7.46 (12)° and the C1—C7—N1—C8 torsion angle is 178.71 (16)°. All bond lengths are within normal values. An intramolecular O2—H2···N1 hydrogen bond (Table 1) is observed and this hydrogen bond produces *S*(6) ring. The O2···N1 separation of 2.569 (2) Å is comparable to those observed for analogous hydrogen bonds in 2-bromo-4-chloro-6-[(*E*)-*p*-tolylimino-methyl]phenol (Zhang, 2009). Molecules are linked into sheets by a combination of O—H···O hydrogen bonds (Table 1). The combination of O—H···O hydrogen bonds generates a chain of edge-fused *R*_6_^6^(22) rings running parallel to the [100] direction (Fig. 2).

### Experimental

The compound (*E*)-2-[(2,4-(dichloro)phenylimino)methyl]-4-hydroxyphenol monohydrate was prepared by refluxing a solution containing 2,5-dihydroxybenzaldehyde (0.03 g, 0.22 mmol) in ethanol (20 ml) and 2,4-dichloroaniline (0.035 g, 0.22 mmol) in ethanol (20 ml). The reaction mixture was stirred for 1 h under reflux. The crystals of the title hydrate suitable for X-ray analysis were obtained from ethanol by slow evaporation (yield 73%; m.p. 432–435 K).

### Refinement

H atoms bonded to O atoms were located in a difference map and refined freely (distances given in Table 1). All other H atoms were placed in calculated positions and constrained to ride on their parent atoms, with C—H = 0.93 Å and *U*_iso_(H)=1.2*U*_eq_(Carrier C).

### Crystal data

**Table:**

C_13_H_9_Cl_2_NO_2_·H_2_O	*F*(000) = 616
*M**_r_* = 300.13	*D*_x_ = 1.517 Mg m^−^^3^
Monoclinic, *P*2_1_/*c*	Melting point: 432 K
Hall symbol: -P 2ybc	Mo *K*α radiation, λ = 0.71069 Å
*a* = 4.6899 (2) Å	Cell parameters from 12355 reflections
*b* = 17.4289 (6) Å	θ = 1.3–27.2°
*c* = 16.1645 (7) Å	µ = 0.50 mm^−^^1^
β = 95.923 (3)°	*T* = 296 K
*V* = 1314.23 (9) Å^3^	Prism, brown
*Z* = 4	0.90 × 0.56 × 0.25 mm

### Data collection

**Table:**

Stoe IPDS II diffractometer	2585 independent reflections
Radiation source: fine-focus sealed tube	1879 reflections with *I* > 2σ(*I*)
graphite	*R*_int_ = 0.050
Detector resolution: 6.67 pixels mm^-1^	θ_max_ = 26.0°, θ_min_ = 1.7°
ω scans	*h* = −5→5
Absorption correction: integration (*X-RED32*; Stoe & Cie, 2002)	*k* = −21→21
*T*_min_ = 0.801, *T*_max_ = 0.959	*l* = −19→19
11982 measured reflections	

### Refinement

**Table:**

Refinement on *F*^2^	Primary atom site location: structure-invariant direct methods
Least-squares matrix: full	Secondary atom site location: difference Fourier map
*R*[*F*^2^ > 2σ(*F*^2^)] = 0.037	Hydrogen site location: inferred from neighbouring sites
*wR*(*F*^2^) = 0.098	H atoms treated by a mixture of independent and constrained refinement
*S* = 0.97	*w* = 1/[σ^2^(*F*_o_^2^) + (0.0595*P*)^2^] where *P* = (*F*_o_^2^ + 2*F*_c_^2^)/3
2585 reflections	(Δ/σ)_max_ = 0.001
188 parameters	Δρ_max_ = 0.14 e Å^−^^3^
0 restraints	Δρ_min_ = −0.29 e Å^−^^3^

### Fractional atomic coordinates and isotropic or equivalent isotropic displacement parameters (Å^2^)

**Table:**

	*x*	*y*	*z*	*U*_iso_*/*U*_eq_	
C1	−0.2010 (4)	0.11322 (10)	0.62421 (11)	0.0455 (4)	
C2	−0.3504 (4)	0.09465 (12)	0.54787 (12)	0.0540 (5)	
H2A	−0.3024	0.0502	0.5207	0.065*	
C3	−0.5665 (4)	0.14033 (12)	0.51181 (12)	0.0556 (5)	
C4	−0.6388 (4)	0.20661 (11)	0.55258 (13)	0.0554 (5)	
H4	−0.7851	0.2380	0.5287	0.066*	
C5	−0.4951 (5)	0.22589 (11)	0.62805 (13)	0.0573 (5)	
H5	−0.5461	0.2702	0.6550	0.069*	
C6	−0.2748 (4)	0.18020 (11)	0.66461 (12)	0.0510 (4)	
C7	0.0230 (4)	0.06279 (11)	0.66080 (12)	0.0485 (4)	
H7	0.0640	0.0180	0.6331	0.058*	
C8	0.3792 (4)	0.03047 (10)	0.76835 (11)	0.0467 (4)	
C9	0.4933 (4)	0.04822 (11)	0.84932 (12)	0.0512 (4)	
C10	0.7030 (4)	0.00366 (12)	0.89192 (13)	0.0560 (5)	
H11	0.7748	0.0162	0.9460	0.067*	
C11	0.8040 (4)	−0.05934 (11)	0.85325 (12)	0.0518 (5)	
C12	0.7014 (4)	−0.07811 (11)	0.77313 (12)	0.0547 (5)	
H12	0.7737	−0.1205	0.7473	0.066*	
C13	0.4907 (4)	−0.03366 (11)	0.73152 (12)	0.0522 (5)	
H13	0.4209	−0.0467	0.6774	0.063*	
N1	0.1649 (3)	0.07852 (9)	0.73022 (9)	0.0492 (4)	
O1	−0.6995 (5)	0.11940 (12)	0.43607 (10)	0.0909 (6)	
H1	−0.848 (8)	0.148 (2)	0.420 (2)	0.121 (12)*	
O2	−0.1365 (4)	0.20076 (10)	0.73902 (10)	0.0729 (5)	
H2	−0.007 (7)	0.166 (2)	0.751 (2)	0.105 (10)*	
O3	−1.1524 (5)	0.19928 (14)	0.37204 (14)	0.0930 (7)	
H1O	−1.167 (7)	0.232 (2)	0.333 (2)	0.112 (11)*	
H2O	−1.317 (10)	0.200 (3)	0.385 (3)	0.154 (17)*	
Cl1	1.06563 (11)	−0.11630 (3)	0.90637 (4)	0.06643 (18)	
Cl2	0.36487 (14)	0.12696 (4)	0.89962 (4)	0.0773 (2)	

### Atomic displacement parameters (Å^2^)

**Table:**

	*U*^11^	*U*^22^	*U*^33^	*U*^12^	*U*^13^	*U*^23^
C1	0.0467 (10)	0.0449 (9)	0.0457 (10)	−0.0009 (8)	0.0091 (8)	0.0010 (7)
C2	0.0598 (12)	0.0552 (10)	0.0472 (10)	0.0099 (9)	0.0062 (9)	−0.0040 (9)
C3	0.0594 (12)	0.0662 (12)	0.0413 (10)	0.0084 (10)	0.0053 (9)	0.0013 (9)
C4	0.0566 (11)	0.0518 (11)	0.0582 (12)	0.0069 (9)	0.0081 (10)	0.0098 (9)
C5	0.0635 (12)	0.0437 (10)	0.0648 (13)	0.0071 (9)	0.0073 (10)	−0.0036 (9)
C6	0.0541 (11)	0.0488 (10)	0.0498 (10)	−0.0028 (9)	0.0045 (9)	−0.0059 (8)
C7	0.0508 (10)	0.0473 (10)	0.0477 (10)	0.0023 (8)	0.0068 (9)	−0.0016 (8)
C8	0.0473 (10)	0.0477 (10)	0.0454 (10)	−0.0023 (8)	0.0065 (8)	0.0031 (8)
C9	0.0502 (10)	0.0521 (10)	0.0508 (11)	−0.0012 (8)	0.0033 (9)	−0.0053 (8)
C10	0.0530 (12)	0.0625 (12)	0.0507 (11)	−0.0034 (9)	−0.0031 (9)	−0.0027 (9)
C11	0.0452 (10)	0.0530 (10)	0.0569 (12)	−0.0043 (8)	0.0030 (9)	0.0063 (8)
C12	0.0592 (12)	0.0493 (10)	0.0565 (12)	0.0044 (9)	0.0107 (10)	0.0009 (9)
C13	0.0607 (11)	0.0525 (11)	0.0432 (10)	0.0021 (9)	0.0046 (9)	−0.0015 (8)
N1	0.0502 (8)	0.0501 (9)	0.0468 (9)	0.0016 (7)	0.0034 (7)	0.0011 (7)
O1	0.0986 (13)	0.1152 (15)	0.0533 (9)	0.0486 (12)	−0.0184 (9)	−0.0210 (9)
O2	0.0780 (11)	0.0689 (10)	0.0678 (10)	0.0162 (9)	−0.0120 (8)	−0.0241 (8)
O3	0.0771 (13)	0.1106 (15)	0.0906 (14)	0.0109 (11)	0.0047 (11)	0.0506 (12)
Cl1	0.0588 (3)	0.0651 (3)	0.0733 (4)	0.0055 (2)	−0.0036 (3)	0.0112 (2)
Cl2	0.0850 (4)	0.0770 (4)	0.0669 (4)	0.0199 (3)	−0.0065 (3)	−0.0257 (3)

### Geometric parameters (Å, °)

**Table:**

C1—C2	1.393 (3)	C8—C9	1.397 (3)
C1—C6	1.398 (3)	C8—N1	1.401 (2)
C1—C7	1.448 (3)	C9—C10	1.380 (3)
C2—C3	1.371 (3)	C9—Cl2	1.734 (2)
C2—H2A	0.9300	C10—C11	1.372 (3)
C3—O1	1.365 (3)	C10—H11	0.9300
C3—C4	1.389 (3)	C11—C12	1.374 (3)
C4—C5	1.373 (3)	C11—Cl1	1.735 (2)
C4—H4	0.9300	C12—C13	1.375 (3)
C5—C6	1.388 (3)	C12—H12	0.9300
C5—H5	0.9300	C13—H13	0.9300
C6—O2	1.354 (2)	O1—H1	0.87 (4)
C7—N1	1.274 (2)	O2—H2	0.87 (4)
C7—H7	0.9300	O3—H1O	0.85 (4)
C8—C13	1.393 (3)	O3—H2O	0.82 (4)
			
C2—C1—C6	118.80 (17)	C13—C8—N1	125.12 (17)
C2—C1—C7	119.88 (16)	C9—C8—N1	117.89 (16)
C6—C1—C7	121.32 (17)	C10—C9—C8	121.82 (18)
C3—C2—C1	121.61 (18)	C10—C9—Cl2	118.39 (16)
C3—C2—H2A	119.2	C8—C9—Cl2	119.78 (15)
C1—C2—H2A	119.2	C11—C10—C9	119.00 (19)
O1—C3—C2	118.39 (18)	C11—C10—H11	120.5
O1—C3—C4	122.44 (19)	C9—C10—H11	120.5
C2—C3—C4	119.16 (19)	C10—C11—C12	121.10 (19)
C5—C4—C3	120.22 (19)	C10—C11—Cl1	119.48 (16)
C5—C4—H4	119.9	C12—C11—Cl1	119.42 (16)
C3—C4—H4	119.9	C11—C12—C13	119.40 (18)
C4—C5—C6	120.91 (18)	C11—C12—H12	120.3
C4—C5—H5	119.5	C13—C12—H12	120.3
C6—C5—H5	119.5	C12—C13—C8	121.69 (18)
O2—C6—C5	119.58 (18)	C12—C13—H13	119.2
O2—C6—C1	121.12 (18)	C8—C13—H13	119.2
C5—C6—C1	119.30 (18)	C7—N1—C8	122.99 (16)
N1—C7—C1	121.34 (17)	C3—O1—H1	113 (2)
N1—C7—H7	119.3	C6—O2—H2	106 (2)
C1—C7—H7	119.3	H1O—O3—H2O	100 (4)
C13—C8—C9	116.98 (18)		
			
C6—C1—C2—C3	0.0 (3)	N1—C8—C9—C10	179.39 (17)
C7—C1—C2—C3	179.05 (17)	C13—C8—C9—Cl2	−179.77 (14)
C1—C2—C3—O1	178.63 (19)	N1—C8—C9—Cl2	0.9 (2)
C1—C2—C3—C4	−0.2 (3)	C8—C9—C10—C11	0.7 (3)
O1—C3—C4—C5	−178.8 (2)	Cl2—C9—C10—C11	179.20 (15)
C2—C3—C4—C5	0.0 (3)	C9—C10—C11—C12	0.5 (3)
C3—C4—C5—C6	0.5 (3)	C9—C10—C11—Cl1	−179.49 (14)
C4—C5—C6—O2	179.88 (19)	C10—C11—C12—C13	−1.0 (3)
C4—C5—C6—C1	−0.7 (3)	Cl1—C11—C12—C13	178.96 (15)
C2—C1—C6—O2	179.87 (18)	C11—C12—C13—C8	0.4 (3)
C7—C1—C6—O2	0.8 (3)	C9—C8—C13—C12	0.7 (3)
C2—C1—C6—C5	0.5 (3)	N1—C8—C13—C12	−179.98 (17)
C7—C1—C6—C5	−178.58 (18)	C1—C7—N1—C8	178.71 (16)
C2—C1—C7—N1	179.43 (17)	C13—C8—N1—C7	9.2 (3)
C6—C1—C7—N1	−1.5 (3)	C9—C8—N1—C7	−171.48 (17)
C13—C8—C9—C10	−1.2 (3)		

### Hydrogen-bond geometry (Å, °)

**Table:**

*D*—H···*A*	*D*—H	H···*A*	*D*···*A*	*D*—H···*A*
O3—H1O···O2^i^	0.85 (4)	1.94 (4)	2.774 (3)	170 (3)
O2—H2···N1	0.87 (4)	1.77 (3)	2.569 (2)	152 (3)
O3—H2O···O1^ii^	0.82 (4)	2.49 (5)	3.184 (3)	143 (4)
O1—H1···O3	0.87 (4)	1.79 (4)	2.659 (3)	171 (3)

Symmetry codes: (i) *x*−1, −*y*+1/2, *z*−1/2; (ii) *x*−1, *y*, *z*.
